# A Non-Adhesive Solid-Gel Electrode for a Non-Invasive Brain–Machine Interface

**DOI:** 10.3389/fneur.2012.00114

**Published:** 2012-07-18

**Authors:** Shigeru Toyama, Kouji Takano, Kenji Kansaku

**Affiliations:** ^1^Biotechnological Rehabilitation Section, Department of Rehabilitation Engineering, Research Institute of National Rehabilitation Center for Persons with DisabilitiesTokorozawa, Japan; ^2^Systems Neuroscience Section, Department of Rehabilitation for Brain Functions, Research Institute of National Rehabilitation Center for Persons with DisabilitiesTokorozawa, Japan

**Keywords:** EEG, BMI, BCI, non-adhesive conductive solid-gel

## Abstract

A non-invasive brain–machine interface (BMI) or brain–computer interface is a technology for helping individuals with disabilities and utilizes neurophysiological signals from the brain to control external machines or computers without requiring surgery. However, when applying electroencephalography (EEG) methodology, users must place EEG electrodes on the scalp each time, and the development of easy-to-use electrodes for clinical use is required. In this study, we developed a conductive non-adhesive solid-gel electrode for practical non-invasive BMIs. We performed basic material testing, including examining the volume resistivity, viscoelasticity, and moisture-retention properties of the solid-gel. Then, we compared the performance of the solid-gel, a conventional paste, and an in-house metal-pin-based electrode using impedance measurements and P300-BMI testing. The solid-gel was observed to be conductive (volume resistivity 13.2 Ωcm) and soft (complex modulus 105.4 kPa), and it remained wet for a prolonged period (>10 h) in a dry environment. Impedance measurements revealed that the impedance of the solid-gel-based and conventional paste-based electrodes was superior to that of the pin-based electrode. The EEG measurement suggested that the signals obtained with the solid-gel electrode were comparable to those with the conventional paste-based electrode. Moreover, the P300-BMI study suggested that systems using the solid-gel or pin-based electrodes were effective. One of the advantages of the solid-gel is that it does not require cleaning after use, whereas the conventional paste adheres to the hair, which requires washing. Furthermore, the solid-gel electrode was not painful compared with a metal-pin electrode. Taken together, the results suggest that the solid-gel electrode worked well for practical BMIs and could be useful for bedridden patients such as those with amyotrophic lateral sclerosis.

## Introduction

The brain–machine interface (BMI) or brain–computer interface (BCI) is a state-of-the art technology that utilizes neurophysiological signals from the brain to control external machines or computers (Wolpaw and McFarland, 2004; Pfurtscheller et al., 2006; Birbaumer and Cohen, 2007). Much effort has been made to help individuals with physical disabilities such as amyotrophic lateral sclerosis (ALS), stroke, or upper cervical spinal cord injury. Non-invasive BMI does not require surgery. Some non-invasive BMI systems make use of hemodynamic signals using functional magnetic resonance imaging (fMRI; Sitaram et al., 2011) or near-infrared spectroscopy (NIRS; Sitaram et al., 2007; Cui et al., 2010), but the majority of papers report methods using electroencephalography (EEG) signals. The P300 speller, a popular BMI system, uses elicited P300 responses to target stimuli placed among row and column flashes (Farwell and Donchin, 1988). We also used EEG signals in a BMI system that enables environmental control and communication using the P300 paradigm (Takano et al., 2009, 2011; Kansaku et al., 2010). Recent studies have evaluated the use of systems relying on these sensory evoked signals of patients with ALS and other diseases (Piccione et al., 2006; Sellers and Donchin, 2006; Ikegami et al., 2011).

Based on BMI studies, it is possible to build an intelligent house in which home electronics or communication tools such as e-mail can be operated with brain waves (Kansaku, 2011). Therefore, we have conducted measurement tests in patient’s homes and in hospitals. However, we felt that the conventional paste-based electrode systems are somewhat awkward to use in practical circumstances (here we use “paste” to mean the conventional paste or gel used for an EEG electrode).

Typical EEG-based BMI electrodes use sticky conductive paste to reduce the impedance between the scalp and electrodes. To use electrodes with paste requires elaborate work not only for preparation but also for removal of the paste. In the preparation stage, paste is first placed on cup electrodes, and then the electrodes are fixed on the head. After using a BMI system, the patient’s head must often be washed to remove the dried paste, which adheres tightly to hair. Similar comments are sometimes found in the literature, and some authors have proposed the use of dry electrodes for BMI to avoid tedious preparation and after treatment (Popescu et al., 2007; Liao et al., 2011; Zander et al., 2011). Popescu et al. (2007) prepared a cap system equipped only with six dry electrodes and a dry reference electrode. The information-transmission rate of their new system was only 30.8% slower than that of their previous experiments using caps with 64 wet electrodes on the same participants. They stated that the advantages of their system were the ease of preparation for EEG measurements and the long-term monitoring capability.

Various types of dry electrodes for EEG measurements have been developed, and one type is a bundle of microneedles (Griss et al., 2001; Chiou et al., 2006; Ruffini et al., 2006; Ng et al., 2009). In this case, microneedles pierce the outer skin layer (*Stratum corneum*), which has high impedance characteristics, and contact the *S. germinativum* layer where living cells exist and are electrically conductive. Therefore, using these electrodes does not require skin preparation. Griss et al. developed such an electrode with a spiked silicon electrode array using a microfabrication technique. Furthermore, Ruffini et al. developed an electrode with an array of multi-walled carbon nanotubes. Although they reported that their electrode was useful, the microneedles must be designed carefully to prevent cell damage.

Conductive textile-based electrodes have been developed for ECG monitoring (Hoffmann and Ruff, 2007; Beckmann et al., 2010). They are comfortable and also durable for long-term use because of their softness. However, these textile-based electrodes are hard to use on hairy sites. Above all, Lin et al. (2011) developed an EEG electrode made of urethane foam covered with a conductive polymer textile. They reported that their electrode can be used on a hairy site.

A popular dry electrode is the pin-based type, which has a metal-pin that contacts the skin. Zander et al. (2011) used a head cap with three pin-based electrodes, a reference electrode, and a ground electrode for their BMI experiments. They concluded that their dry electrode system showed no degradation in EEG and BMI performance in most cases. Liao et al. (2011) also used a metal-pin electrode system. They obtained similar brain waves from both dry and wet electrodes in simultaneous measurements. Sellers et al. (2009) compared pin-based and paste-based electrodes by simultaneously mounting these electrodes on a specially developed headpiece. The EEG signals of these two types of electrodes were almost identical, and the BMI classification accuracy was also almost identical while performing a copy-spelling task. Although these dry electrodes have been reported to be useful, we are concerned that they may cause pain or may injure the surface of the patient’s head when hard solid electrodes are used, particularly when the patient is lying on a bed with his/her head resting on the pin electrodes.

We have developed several types of electrode for BMI (Toyama et al., 2011a,b). In this study, we prepared a specially designed conductive solid-gel as an electrode material for BMI. The solid-gel retains moisture and is not sticky like paste (here we use “gel” as a chemically defined term). We selected the ingredients carefully to produce a moisture-retaining solid-gel. The solid-gel contained a liquid comprising carboxymethylcellulose (CMC), CaCl_2_, glycerol, and pure water. The superior wettability of our solid-gel was attributed to the nature of the ingredients. CaCl_2_ and glycerol are water-absorbing materials. The weight of these materials increases when they are exposed to a normal room environment. Due to an electrochemical reaction, a combination of Ag/AgCl electrodes and KCl-containing liquid or solid-gel is usually recommended as an electrode material to measure biopotentials. The potential between the electrode and solution in contact with that electrode is constant for a long period. By contrast, the electrode signal is apt to drift in the long-term when we use non-recommended materials, such as CaCl_2_. However, this problem has essentially been alleviated with modern circuit technology. Today, we have extremely high-precision analog-to-digital converters (ADCs) for medical research and clinical measurement systems (Aksenov et al., 2001). For example, when a 24-bit ADC is used to capture the voltage signal, a signal with 0.12 μV precision and ±1 V full range can be obtained, which is sufficient to cover the drift due to the electrochemical reaction at the electrode surface. Therefore, operators of the measurement system can use digital filters to subsequently obtain brain waves.

Here, we examined the usefulness of a new system with the in-house conductive solid-gel-based electrode by comparing it with in-house metal-pin-based electrodes and conventional paste-based electrodes.

## Materials and Methods

### Solid-gel electrode

**Figure 1:**
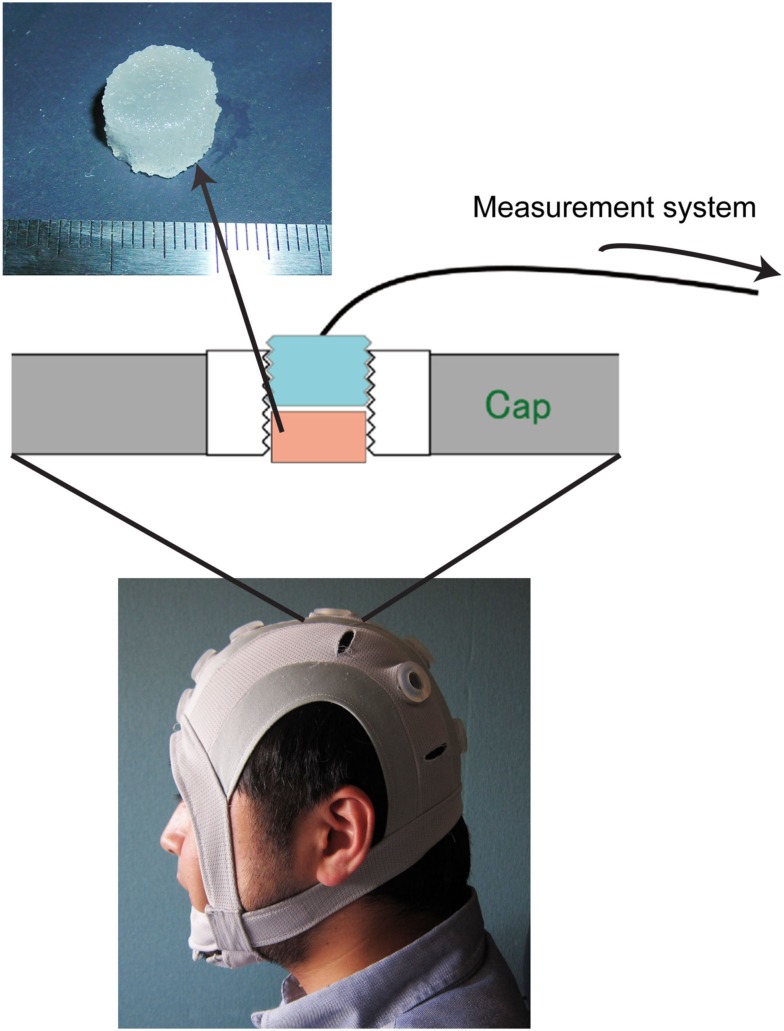
**Photograph of the originally developed head-mounted cap, which has 12 electrode sockets**. One of the electrode sockets on the cap is enlarged to explain how the solid-gel is used. The solid-gel chip is loaded in the through-hole; the hole is then plugged with an electrode plate. The diameter of the chip was approximately 12 mm, and the height was approximately 7 mm.

The solid-gel chip (Figure 1) was made of CMC sodium salt (MW, 700 kDa; Sigma Chemical, St. Louis, MO, USA), calcium chloride dihydrate (Wako Pure Chemical Industries, Ltd., Osaka, Japan), glycerol (Nakalai Chemicals, Kyoto, Japan), and pure water. The solid-gel contained 10.9, 38.0, 7.6, and 43.4% of these compounds by weight, respectively.

### Metal-pin electrode

**Figure 2:**
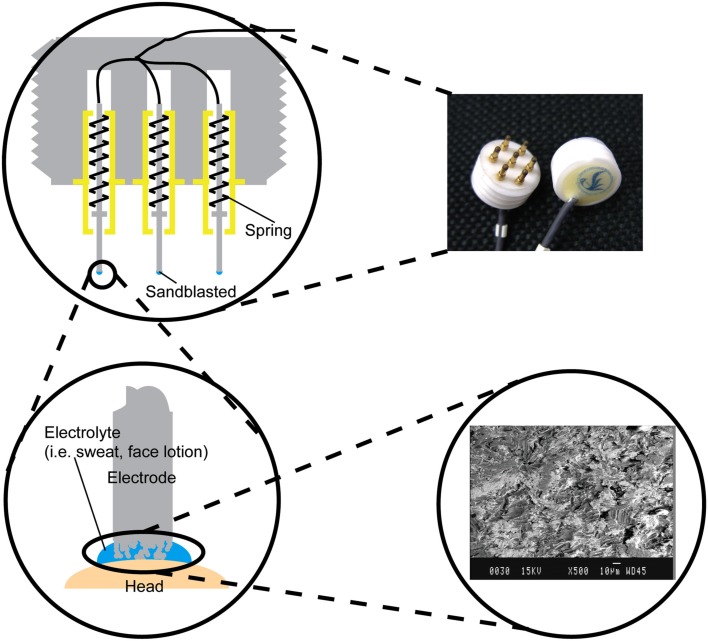
**Photograph of the metal-pin electrodes and illustration of the electrode structure**. The electrode has seven retractable pins. The tip of each pin was sandblasted to enlarge the effective surface area (see inset scanning electron micrograph).

The structure of the metal-pin electrode is shown in Figure 2. Seven metal-pins extended from one side of a cylindrical main body, which was made of epoxy resin. Each metal-pin was composed of a center rod, an outer sheath, and a spring. The rod could be forced inward under pressure, so it was harmless to the scalp. The effective area of the rod tip was enlarged by sandblasting to decrease the contact impedance between the rod and the skin. Additionally, a wire was fixed at the end of the rod instead of at the outer sheath to minimize the friction noise caused by sliding between the center rod and the outer sheath.

### Commercialized paste

In this experiment, we also used a conventional paste (ABRALYT 2000; abrasive electrolyte-gel; EASY CAP, Munich, Germany) as a control material to compare its usefulness.

### Head-mounted cap

We developed an original head-mounted cap with 12 electrode sockets (Figure 1) for use with solid-gel electrodes, metal-pin electrodes, and commercial paste. Each electrode socket had a silicone-based grommet equipped with a through-hole where the solid-gel-based, metal-pin, and paste electrodes were inserted. During EEG measurements, one electrode was used as a reference. The diameter and depth of the through-hole were 15 and 8.5 mm, respectively. When using a solid-gel chip, the chip was inserted into the through-hole of the grommet, which was plugged with an electrode plate. The body of the electrode plate was made of epoxy resin and was cylindrical in shape, and its outer wall had concentric grooves. The inner side of the through-holes of the cap had concentric grooves with the same pitch as that of the plate, so that the plate was held tightly in the through-hole. A flat Ag/AgCl plate was attached to the bottom of the plate, and an electric wire protruded from the rim of the top. The electric wire was solder-mounted on the Ag/AgCl plate at the inside of the plate and penetrated it. Wire terminals were connected to the brain-wave-measurement system.

### Measurement of volume resistance: Solid-gel vs. paste

The volume resistance of the solid-gel and paste was evaluated using a rectangular measurement cell in which the facing sides were a pair of plain electrodes. The cell was filled with sample material, and its impedance was evaluated with a frequency response analyzer (S-5720B, NF Corp., Kanagawa, Japan).

### Evaluation of viscoelasticity: Solid-gel

The dynamic viscoelasticity of the solid-gel was evaluated using a rheometer (NDS-1000, Taisei, Saitama, Japan). The complex modulus of a sample was obtained from the dynamic displacement response on applying sinusoidal pressure (3 Hz). The dynamic viscoelasticity of a silicone rubber plug (E-02, Taiyo Kogyo, Tokyo, Japan) was also measured as a reference sample.

### Evaluation of moisture-retention property: Solid-gel vs. paste

The moisture-retention property of the samples was evaluated by measuring their weight under a controlled atmosphere. Each sample filled a small cylindrical plastic cup (diameter: 19 mm; depth: 22.5 mm) to the top. Then, the cups were put into a thermo-hydrostat (TPAV-48-20, ISUZU, Tokyo, Japan) without a cover, and the cups were weighed every 2 h. Temperature and relative humidity were constant at 23°C and 40%, respectively. Two kinds of liquid sample were prepared to contrast the moisture-retention property of the solid-gel and the conventional paste. One liquid contained 42.7, 8.5, and 48.7% of CaCl_2_·2H_2_O, glycerol, and H_2_O by weight, respectively. The other liquid was a 3 M KCl/H_2_O solution.

### Impedance evaluation: Solid-gel vs. metal-pin vs. paste

The participants in the electrode impedance evaluation were 11 healthy adults (six males, five females; age range 20–39 years; average 28.2 years). The experiment was approved by the Institutional Review Board, and all participants provided written informed consent according to institutional guidelines. The impedances of the solid-gel, metal-pin, and conventional paste electrodes were measured simultaneously by positioning these electrodes at P3, Pz, and P4, respectively.

### BMI measurements: Solid-gel vs. metal-pin

For the P300-BMI tests, four healthy males (age 26–39 years) were recruited as participants. The experiment was approved by the Institutional Review Board, and all participants provided written informed consent according to institutional guidelines. The participants were required to input 15 hiragana characters from the Japanese alphabet using a row and column flicker panel with an 8 × 10 matrix in each condition. For this purpose, we modified the P300 speller (Farwell and Donchin, 1988), which uses the P300 paradigm, which presents a selection of icons arranged in a matrix. The participant focuses attention on one of the icons in the green/blue flicker matrix as a target, and each row/column of the matrix is intensified in a random sequence. The target stimuli are presented as the rare stimuli (oddball paradigm). P300 responses to the target stimuli were elicited, and the extraction and classification of these responses can be used to get the target. One complete cycle of eight row intensifications and 10 column intensifications constitutes a sequence. Online performance was evaluated, and each letter was selected in a series of 10 sequences. Eight-channel (Fz, Cz, Pz, P3, P4, Oz, PO7, PO8) EEG data were recorded using an in-house cap and an amplifier (g.USB amp, Guger Technologies, Graz, Austria). All channels were referenced to the Fpz, and grounded to the AFz electrode. Fisher’s linear discriminant analysis was used for classification purposes. The details of experimental setting were same as in our previous study (Takano et al., 2009).

## Results

**Figure 3:**
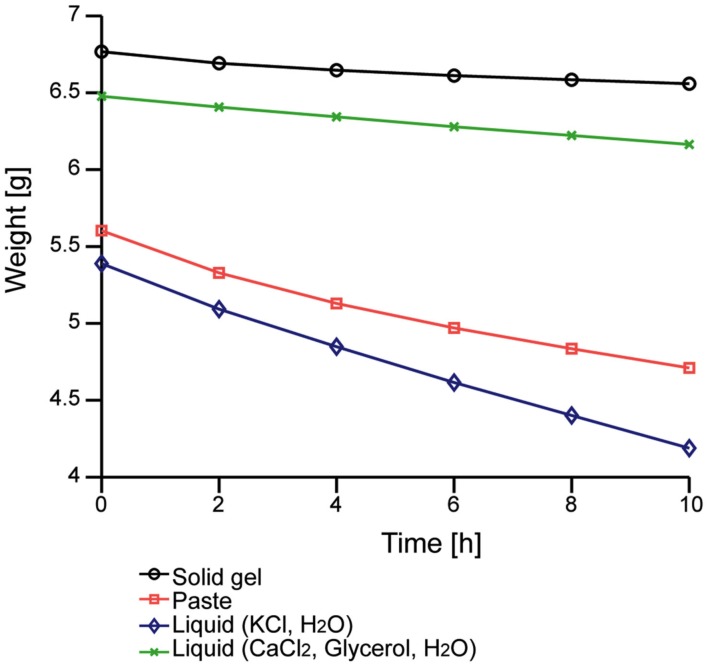
**Time-dependent changes in the weight of the solid-gel and other materials**. Data of four materials were taken simultaneously.

We first evaluated the moisture-retention property. Figure 3 shows the time-dependent change in the weight of our solid-gel. The decrease in the weight of the liquid, which was the electrolyte of the solid-gel, was less than that of the KCl solution that is normally used for conventional electrodes. The decrease in the weight of the solid-gel material was also smaller than that of conventional conductive paste, suggesting that the solid-gel remained wet for more than 10 h in the dry environment.

The fabricated solid-gel chips were not sticky, but elastic. The complex modulus was 105.4 kPa, whereas that of the silicone rubber plug was 2088 kPa. Moreover, the volume resistivity of the solid-gel was about 13.2 Ωcm, which is sufficiently low to obtain brain waves. For comparison, the volume resistivity of the conventional paste was measured as 64.8 Ωcm. Before inserting the solid-gel chips into the through-holes of the head-mounting cap, we pushed aside the hair appearing at the bottom of the through-holes. However, this process was not laborious when we became accustomed to it, and it took 30–60 s for each chip setting, even in the worst case. The solid-gel chip was deformed when it was inserted into the through-hole and exposed to pressure from pushing the electrode plate with a finger, thereby penetrating the hair mesh and attaching to the scalp skin surface. The total setting time required before the measurement was 5–10 min.

**Figure 4:**
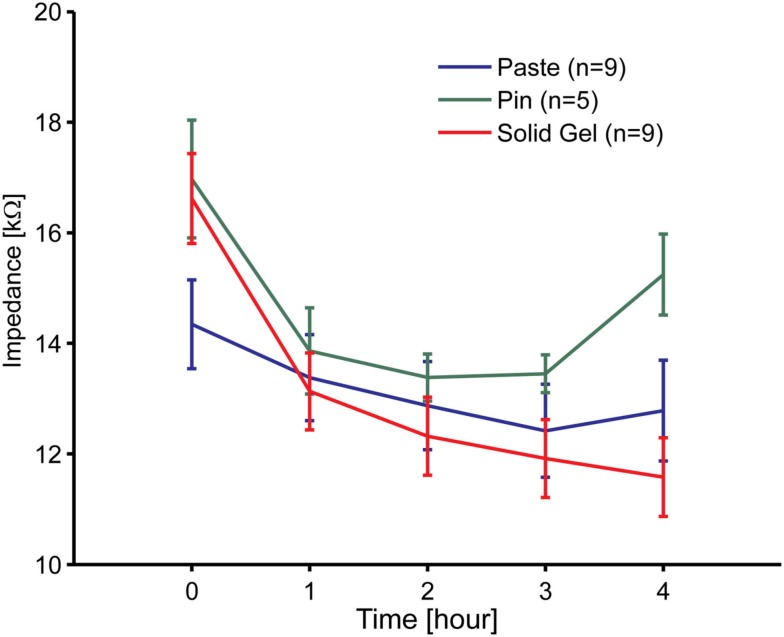
**Time course of the electrode impedance of the solid-gel, metal-pin, and conventional paste-based electrodes mounted on the head**. Nine participants participated in these experiments at most. However, data for two participants using to the paste-based electrode was omitted, because the values were abnormally high due to drying.

An impedance evaluation test was carried out to evaluate electrode attachment. Figure 4 shows the impedance time course between the reference and the brain-wave collecting electrodes including the newly developed electrodes. Although there were 11 participants, some data were omitted. The data from two females were omitted because the impedance of the paste-based electrode abnormally increased during the measurement. The impedance for one person was 92 kΩ at 3 h and 116 kΩ at 4 h, and that of the other person was 468 kΩ at 3 h and 5654 kΩ at 4 h. In these cases, we noted that the paste had dried. Data for the metal-pin electrodes in six individuals were also omitted due to irregular impedance values from the start; the main reason was likely the thick hairs between the electrode and scalp, which would cause poor contact between them. Therefore, data of nine participants (six males, three females) for the paste- and gel-based electrodes and five participants (four males, one female) for the metal-pin electrodes remained.

The initial impedance of the solid-gel-based electrode was slightly higher than that of the paste-based electrode, but it decreased gradually and was sufficiently low within 1 h. The impedance ranged from 3 to 25 kΩ (typically 10 kΩ). Moreover, it was noticeable that only the impedance of the solid-gel-based electrode decreased continuously. In two cases, we continued impedance measurements with the solid-gel-based electrode and discovered that the impedance was almost constant for at least 9 h in both cases (data not shown). Furthermore, from the data for the five participants in whom we evaluated the impedance with all three electrode types, ANOVA and the *post hoc* Tukey–Kramer test revealed that the impedance (at 4 h) of the metal-pin electrode was significantly higher than those of the other electrodes [*F*(2,8) = 12.01, *p* = 0.0039]. A similar tendency in the impedance behavior was observed with a slightly different solid-gel containing MgCl_2_ instead of CaCl_2_ (data not shown).

**Figure 5:**
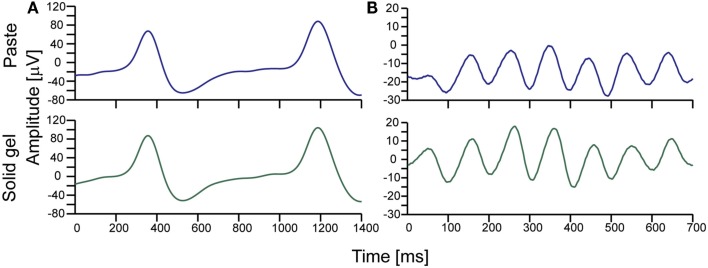
**Typical brain waves observed during simultaneous measurement using the solid-gel- and paste-based electrodes**. **(A)** Brain waves observed during intermittent eye blinking. **(B)** Alpha waves. The upper and lower waves in each graph correspond to the signals obtained with the paste-based and solid-gel-based electrodes, respectively. The signals were collected with an in-house EEG amplifier (24 bit, 1024 Hz). The represented data were obtained through an eighth-order bandpass filter (1–15 Hz).

After checking electrode impedance, we successfully obtained brain waves with the solid-gel-based electrodes. As shown in Figure 5, we observed similar α-waves and the spike-like waves corresponding to eye blinking during the measurements using the solid-gel- and paste-based electrodes.

**Figure 6:**
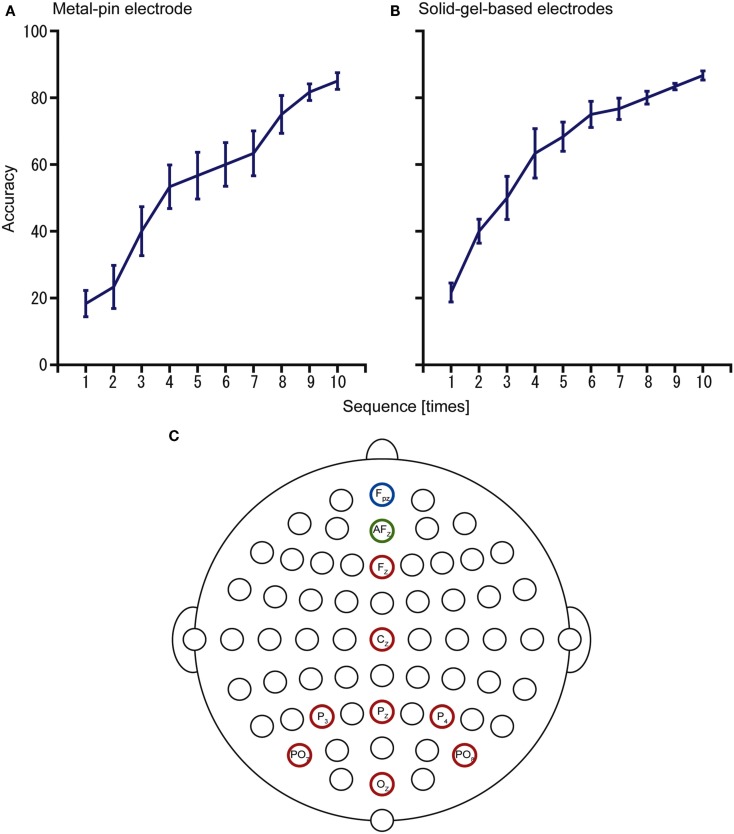
**Results of the P300-brain–machine interface (BMI) experiments using (A) metal-pin electrodes and (B) solid-gel-based electrodes**. The configuration of electrodes is represented in **(C)**. Data were obtained from the same participants (*n* = 4).

Furthermore, we examined the use of the electrodes for operating a P300-BMI to input hiragana characters (Figure 6). With the metal-pin electrode, the mean accuracy was 85% (*n* = 4, 73.3–92.3%), whereas with the solid-gel-based electrode, the mean accuracy was 86.7% (*n* = 4, 80–92.3%). These results showed the potential of these electrodes for practical use as a BMI system in individuals with disabilities.

The conventional paste was apt to dry, especially peripherally, where it was exposed to the atmosphere; it was also difficult to remove without washing the hair after use. By contrast, the solid-gel-based electrodes had superior wettability throughout the extended measurements. The solid-gel remained wet for more than 9 h after the measurement; consequently, it was easy to remove, and almost no residual solid-gel was seen after use. Moreover, we observed no skin problems after using the solid-gel-based electrodes in our experiments.

Our solid-gel chips can be stored for more than 3 months at room temperature without any significant change when packed in a sealed container, suggesting the practical usefulness of our solid-gel chip.

## Discussion

We prepared a conductive solid-gel-based electrode and a metal-pin-based electrode. An examination of the weight change in the solid-gel material under a controlled atmospheric environment revealed superior retention of wettability by our solid-gel compared to that of conventional paste. The electrode impedance measurements were comparable between the solid-gel-based electrode and the conventional paste-based electrode, whereas the impedance of the metal-pin electrode was higher than that of the newly developed electrodes. Furthermore, we obtained almost equivalent signals with the solid-gel-based and paste-based electrodes during simultaneous brain-wave measurements. Finally, we successfully performed P300-BMI using both the metal-pin and solid-gel-based electrodes.

The solid-gel is elastic and is soft enough for use on human skin. Boyer et al. (2009) reported that the complex modulus of the skin on the arm is 10.7 ± 2.64, 8.09 ± 1.84, and 7.17 ± 2.06 kPa for participants 18–30, 31–50, and 51–70 years old, respectively. The complex modulus of the solid-gel was 105.4 kPa, whereas that of silicone rubber was 2088 kPa. Although the scalp is slightly harder than the arm skin, the silicone rubber was much harder than either of these.

All ingredients used in our solid-gel are food additives. CMC is used as a thickener in ice cream (Regand and Goff, 2002), and glycerol is used as a sweetener or moisturizer. Calcium chloride is used to prepare a coating material on fruit (Oms-Oliu et al., 2010). In East Asian countries such as Japan, calcium chloride is also used to coagulate soy solution to produce tofu (bean curd; Prabhakaran et al., 2006). Furthermore, one of the main reasons for adopting calcium chloride was that some commercial EEG pastes already contain calcium (for example, Weaver and Co., Ten20 Conductive Paste; Nihon Kohden, Elefix). Although calcinosis has been reported after using a calcium-containing electrode paste (Wiley and Eaglstein, 1979; Mancuso et al., 1990; Puig et al., 1998), we have not seen unfavorable cases in our experiments. However, we prefer magnesium chloride to calcium chloride for future work because we obtained similar results from the electrodes using either of them in preliminary testing.

In a textbook on EEG (Niedermeyer and Silva, 1999), the recommended contact impedance between the electrode and skin is <5 kΩ, whereas that of our solid-gel was about 10 kΩ. Although the impedance of our solid-gel-based electrode seems slightly higher than the recommended value, it is acceptable because the impedance depends on geometry and electrode size. In our case, the paste-based electrode with the same geometry and size as those of the solid-gel electrode had similar value. Compared with the dry electrodes in the literature, the contact impedance of the solid-gel-based electrode was comparable or better. The reported impedance is 4–26 kΩ with a polymer form electrode (Lin et al., 2011), <20 kΩ with a bundle of pin electrodes (Zander et al., 2011), and 7–25 kΩ with arrayed spike electrodes (Ng et al., 2009). In our experiments, the impedance of the metal-pin electrode was about twice as high as that of the solid-gel-based electrode.

We successfully obtained brain waves with the BMI system employing a solid-gel-based electrode. The signals were almost equivalent to those observed with conventional paste-based electrodes. Moreover, the electrode does not require elaborate preparatory work or removal of paste adhered to hair and skin after measurement.

We have already revealed the usefulness of our BMI system using conventional paste-based electrodes (Takano et al., 2009; Ikegami et al., 2011). Carrying out a similar test of P300-BMI, we showed the usefulness of our newly developed metal-pin and conductive solid-gel-based electrodes here. The solid-gel-based electrode is soft and less harmful to scalp skin, whereas pain was sometimes felt with the metal-pin electrodes. Most users will be bedridden individuals such as patients with ALS; therefore, the soft solid-gel-based electrode is a good first choice for that purpose. Furthermore, our developed electrodes can be used for other long-term EEG investigations such as sleep recordings, anesthesia monitoring, intensive-care monitoring, and long-term monitoring of patients with epilepsy (Niedermeyer and Silva, 1999).

## Conflict of Interest Statement

The authors declare that the research was conducted in the absence of any commercial or financial relationships that could be construed as a potential conflict of interest.
